# Integrating socio-cultural value system into health services in response to Covid-19 patients’ self-isolation in Indonesia

**DOI:** 10.1057/s41599-023-01629-7

**Published:** 2023-04-12

**Authors:** Yety Rochwulaningsih, Singgih Tri Sulistiyono, Mahendra Puji Utama, Noor Naelil Masruroh, Fanada Sholihah, Fajar Gemilang Purna Yudha

**Affiliations:** 1 Departement of History, Faculty of Humanities, Universitas Diponegoro, Semarang, Indonesia; 2 Faculty of Medicine, Universitas Diponegoro, Semarang, Indonesia

**Keywords:** Sociology, History

## Abstract

This article studies the synergistic sociocultural value system to handle COVID-19 patients in self-isolation in Indonesia, to find an effective formula in COVID-19 spread control. The problems studied here is the response carried out and the constraints faced by the Indonesian government related to the self-isolation policy. Why and how does the Indonesians’ sociocultural value system contribute to COVID-19 patient response? Through the survey conducted via Google Forms, in-depth interviews, focus group discussions and literature study, it is found that the Indonesian government issued the self-isolation regulation for COVID-19 patients to mitigate the rapid and massive COVID-19 transmission. However, many constraints are found in the policy implementation, including; people’s insufficient knowledge and understanding of COVID-19, leading to negative implications for the COVID-19 survivors or patients’ perception, causing social stigmatisation for COVID-19 survivors or patients; COVID-19 task force’s limited access to medicine, medical instrument and hospital facilities. Meanwhile, the Indonesians’ strong socio-cultural values like tolerance, mutual aid, and communal work, including among the educated people in urban society, may be potentially integrated into the health service to respond to COVID-19 patients with their self-isolation. Therefore, their integration and empowerment can be a solution to mitigating COVID-19 transmission in Indonesia.

## Introduction

After the World Health Organization (WHO) on 11 March 2020 announced Coronavirus Disease 2019 (COVID-19) as a global pandemic, since it had spread to 118 countries and infected more than 121,000 people in Asia, Europe, Middle East and the US with a significantly increasing trend until early September 2021 with up to 218,946,836 confirmed positive Covid-19 cases and 4,539,723 deaths, various response efforts had been conducted almost in all countries, including Indonesia. In this context, since the beginning, Indonesia had taken various policies related to clinical management, infection control, specimen management and laboratory confirmation, risk communication and empowerment. Even after the number of people positively exposed to COVID-19 kept increasing significantly, the government, through the Ministry of Health of the Republic of Indonesia issued technical guidelines on the examination, tracing, quarantine, and isolation in order to accelerate COVID-19 prevention and response as stated in Decision of Minister of Health of the Republic of Indonesia No. HK.01.07/Menkes/4641/2021 dated 11 May 2021. However, the implementation had not shown the expected results where the number of people positively confirmed with COVID-19 kept increasing with a high death rate, taking 13th place in the world, with 4,116,890 confirmed positive COVID-19 cases and 134,930 deaths (WHO Coronavirus COVID-19 Dashboard (2021)).

The COVID-19 pandemic has changed people’s social-economic life pillars. Still, there are only a few researches and publications on that issue, especially related to the response to COVID-19 patients in self-isolation. Those who carried out self-isolation, commonly among the rationalists, faced big problems since they should live at home, cease working, and had no income (those who do not have permanent income) and could not directly interact with their social environment. Even according to the results of research in Semarang, Central Java, Indonesia, COVID-19 brought significant impacts on occupations, in the form of delayed, declined and even lost work (Amirudin et al. 2021). In Central Java, the Governor Ganjar Pranowo announced *Jogo Tonggo* [taking care of the neighbours] programme that is deemed effective in helping food resilience, accelerating the information rate of COVID-19 response and education, stimulating human moral awareness to abide by regulations and creating solidarity for protecting each other and fulfilling neighbours’ needs so as to improve the immunity of COVID-19 patients and their family so that COVID-19 patients’ recovery rate increases (Shodiq, 2021). However, the research shows that only a small part of the people is aware of and implementing the Provincial Government of Central Java’s programme and in general about 9% participants are not aware of such a programme from the Regional Government in Indonesia. About 81.1% paid attention and gave assistance to those in self-isolation more because of the sense of social obligation, and the remaining because of kinship, close neighbour and a close friend relationship. Thus, they are moved to help those exposed to COVID-19.

The manifestation of a functioning sociocultural value system in helping COVID-19 patients in self-isolation is reflected, besides the involvement of governmental institutions such as the COVID-19 Task Force, also the trending various social movements, both initiated by the Indonesians individually or in groups that is based on primordial and personal relationships such as blood relationship, region of origin, friendship; formal and informal, etc. as the indicators that the Indonesians’ sociocultural value system is still strong. This is the social practice reproduced by an individual or group that could be a social capital in solving unexpected strategic problems that are greatly affected by the level of the social bond built (Giddens, 1984: 17). In Yogyakarta, on micro-scale, there is a social movement by the Jogja Food Solidarity (*Solidaritas Pangan Jogja*/ SPJ) and the COVID-19 Response Inter-Faith Network (*Jaringan Lintas Iman Tanggap COVID-19*/ JIC) that voluntarily helped people who are exposed to COVID-19 (Haryadi & Malitasari, 2020) In Semarang, a ‘COVID-19 Shelter’ gave free aid to those in self-isolation. In addition, some online applications such as #SemarangbisalawanCOVID-19 and, in Indonesia, #indonesiabisalawanCOVID19, #wargabantuwarga.com, etc.

The concrete form of assistance given by the public, either individually or by the organisation, is mostly (62%) foods, drinks and fruits, while the next highest percentage is of medicine, vitamin, supplement, herbal medicine and the next are basic necessities and others. The interesting thing about this research is that the various assistances are mostly (51.5%) given directly by the givers to the self-isolation location, and the next percentage is coordinated and scheduled in turn by end environmental administrators using courier service. All of which are carried out while applying the health protocol in an orderly and disciplined way, that is placing them in places provided outside of the house (table or chair on the terrace or fence). This might be related to the good knowledge of COVID-19 and the sociocultural value of assistance givers. Although most of the participants (77.3%) lived in a rural area with high educational level (Senior High School 25.4%; S1 29.2%; S2 25.5; and S3 9.4%) distributed throughout Indonesia, they have high social concern that is realised through the various assistances given to COVID-19 survivors/patients in self-isolation. This is in line with the opinion of Wiepking & Maas (2009) that education is an important element in growing a better understanding of the complexity of issues. They generally underwent an internalisation and socialisation process of sociocultural values, covering tolerance, mutual aid, and communal work, mostly of the agrarian cultural value system (Wolf, 2004). In addition, social status is also correlated with the chance and opportunity to give social assistance to others.

Differently from Indonesia, in German and some other countries, self-isolation is defined more as social isolation and deemed as the form of alienation of COVID-19 survivors/patients whose basic rights of descent and social life are seized. Such a condition triggered local social solidarity to assist the survivors. The motive of giving assistance could not be separated from the purpose of obtaining assistance when they face the same. Assistance is not only given to family, but also to neighbours and friends, and even non-acquainted people. Therefore, COVID-19 could strengthen solidarity or start building new social capital (Koos & Bertogg, 2021). It is different from the situation in Indonesia where social concern, mutual aid and communal work had rooted in the Indonesians’ sociocultural value system, and it is not a new social system that is formed by the COVID-19 pandemic. Therefore, this paper further discusses the sociocultural value system, especially related to self-isolation procedures conducted by COVID-19 patients in Indonesia. It also observes the constraints and effectiveness of the sociocultural value system rooted in the Indonesians.

## Methodology

This qualitative research is built from the assumption that the reality of the social solidarity movement for COVID-19 patients in self-isolation in Indonesia is the result of sociocultural construction formed through inter-individual, inter-group and individual-group interactions (Berger & Luckmann, 1990: 1; Bogdan, B. (1982)). In this context, understanding social reality from the actors’ perspective as the research subject is quite important to catch the meaning behind their inter-individual, individual-group and inter-group interactions (Denzin & Lincoln, 2005). The data are collected through surveys, in-depth interviews, focus group discussions, and literature studies. Due to the COVID-19 pandemic, surveys are collected through Google Forms. Furthermore, in-depth interviews and focus group discussions are conducted through Zoom Meetings.

### Participants profile

The survey was carried out on 2–27 July 2021, while Focus Group Discussion (FGD) was conducted on 9 August 2021, and in-depth interview on 9–15 August 2021. The survey was collected through Google Forms. The participants are determined through purposive sampling considering the Indonesian appropriateness aspect (Patton, 2002). This survey is intended for survivors of COVID-19 who have undergone self-isolation and people who have never been infected with the COVID-19 virus yet have experience carrying out solidarity actions. Participants provided their age, gender, province of residence, education, occupation, and various types of geographic locations (urban, rural or agrarian, and coastal). Furthermore, participants should respond to questions regarding self-isolation, namely: types of treatment; medical assistance from the government through village midwives or primary healthcare (Puskesmas) and the Covid-19 Task Force; activities to help residents undergoing independent isolation; how to provide this assistance to residents experiencing self-isolation; and reasons for participating in assisting people who are self-isolating. There are 445 (276 or 62% of women) participants from 29 provinces in Indonesia, including Sumatra, Java, Bali, Kalimantan, Sulawesi, Maluku, Nusa Tenggara and West Irian with educational background from elementary school (1), junior high school (1), senior high school (113), bachelor (130), master (158), doctoral (42). The age of our sample ranged from 18 to 74 years old. The occupations of the participants are diverse, including employee from Non-Government Organization (NGO) (6 or 1.3%), state-owned enterprises (6 or 1.3%), entrepreneurs (31 or 7%), private employees (86 or 19.3 %), government employees (118 or 26.5%), and others (131 or 29.4 %). Furthermore, the majority of participants are from urban (344 or 77,3%), rural or agrarian (86 or 19.5%), and the rest are from coastal areas (15 or 3.4%).

### Data analysis

The data were treated as anonymous. Data analysis was used to classify and compare survey results based on age, gender, regional geographic range (city and province). Meanwhile, Kruskal-Wallis with Chi-Square test to determine significance rate statistically difference between two or more independent variables which scales into numerical data (interval/ratio) and ordinal scale. Statistical data analysis has been corroborated with information from in-depth interview and Focus Group Discussion (FGD) or simultaneously with data collection process in an interactive model analysis, that is to make taxonomy category classification, data interpretation in matrix, and formulation of points of conclusion (Miles & Huberman, 1984). Meanwhile, interviewees were selected due to their experiences in handling the patient’s isolation cases. For instance, those who are working on primary healthcare and community figures who were engaged in solidarity actions.

## Self-isolation policy for COVID-19 patients

Starting from the fact that COVID-19 is a contagious disease caused by the SARS-CoV-2 virus and has been defined as a global pandemic by WHO, of which transmission situation at international and national levels is still relatively high, the response effort is conducted through various methods. Besides the increasing number of cases and death tolls because of this pandemic, there are also significant social, economic, political, and psychosocial impacts (Banerjee, 2020: 66). It is assumed that almost four billion people are living in isolation during this pandemic (Matias et al. 2020: 25). Billions of people are quarantined at home because many countries applied lockdown in order to enforce social distancing as the measure to restrain the infection, confirmed cases and isolated suspects. Therefore, strategic measures are needed to trace, quarantine, and isolate COVID-19 cases in order to accelerate its prevention and control.

Examination, tracing, quarantine, and isolation are a process of a set of continuous activities that will be successful if carried out quickly and with discipline. Therefore, this process needs public involvement in its implementation and coordination between governmental units at various levels (Kementerian Kesehatan Republik Indonesia, 2021). According to the Indonesian Ministry of Health, by the mean incubation period of COVID-19 (the time when an individual is infected until the emergence of symptoms) is 5–6 days, even if, in a few cases it can be up to 14 days and because of unavailable adequate resources, self-isolation must be conducted for 14 days.

An infected individual can be a transmission source from about 2 days before they show any symptoms. The COVID-19 incubation period is the basis of consideration for examination, tracing, quarantine, and isolation strategy. This strategy can also be sharpened using information from laboratory results. This research exposes an interesting point, since in line with the government’s provisions, those who are in self-isolation according to the swab test reactive/positive are 62.1%, then followed by symptoms like heat, fever, cough is 28.5%, and the rest 9.4% identified by contacting with COVID-19 patients. Self-isolation is pursuant to the provisions also implemented with coordination and monitoring by relevant officers, namely COVID-19 Task Force, involving health, security, police, and governmental bureaucratic institutions until the lowest level institution (*Rukun Tetangga*/ neighbourhood association).

Self-isolation or home care is carried out for people with mild symptoms and without accompanying conditions such as (lung, heart, kidney disease and immunocompromised conditions). The isolation can be performed on patients under surveillance, people under monitoring and close contacts who are symptomatic while still paying attention to the possibility of worsening. Some of the reasons patients are treated at home are that inpatient care is unavailable or unsafe. Such considerations must take into account the clinical condition and environmental safety of the patient. Location considerations can be made at home, in public facilities, or by means of transportation, considering local needs and situations (Kementerian Kesehatan Republik Indonesia (2020): 63).

The research result shows that most of the participants (59.1%) are aware of the coordination and monitoring by governmental institutions and are even given medication assistance by the government. Although the series of self-isolation activities conducted in preventing and controlling COVID-19 had operated, some problems still arose in the community besides the ones related to a limited number of medical workers and supporting facilities and infrastructure, no less important is the issue of social stigma on COVID-19 patients/ survivors.

## How is social solidarity understood by the community?

Social solidarity arose in response to the similar feeling for the covid-19 global pandemic, allowing individuals to take real action to solve difficulties that others face. Adoption of the conceptual framework can be based on Robert Campbell et al. ‘s theory, that social solidarity and collective action can reflect an individual’s action and attitude implementation related to trust, altruism, and feedback. This framework can be used to explore an individual’s action in reflecting or predicting what may occur in a collective situation, in this case like social solidarity in strengthening and supporting covid-19 patients in self-isolation. The social solidarity theory, according to Durkheim, can be reflected in reducing social distance, social expulsion, and stigma arising from health risk, prejudice, fear, and rumour which damage social integration (Mishra & Rath, 2020). The essential factor of solidarity and social concern is cohesion between individuals in the community to ensure social order and stability. Therefore, there is a feeling of inter-individual dependence in a community, enabling them to improve others’ lives (Durkheim, 1933). Individuals must consider not only their interest, but also their obligations to the community and collective responsibility to improve public welfare in general (Paskov & Dewilde, 2012), thus stimulating concern (Reichlin, 2011).

In the context of COVID-19 pandemic that struck almost all countries in the world, the people at risk of infection feared death, non-welfare, expulsion, and change in lifestyle, causing psychological pressure on every individual. The phenomenon occurred almost in all countries in the world, including Indonesia. Consequently, the people seemed emotionally fatigued during the pandemic since they could not relieve their emotional burden during self-isolation. Self-isolation and restriction activities in other forms are thus often deemed as an effort to violate human rights (Jeffrey, 2020). The studies conducted by Rahman et al. (2021) and Anindyajati et al. (2021) show that restriction in the form of self-isolation and other restricted activities has caused excessive anxiety in society at a proportion of 14.8% as may be identified in some factors, namely: fear of expected exposure to virus, false interpretation in identifying body signals indicated as infection, an increasing number of sources of information from social media, online newspapers, television news, causing panic and worry (Anindyajati et al. 2021; Rahman et al. 2021). This mental condition may influence physical health. Thus, rules are needed to control the validity and reliability of the information given by the media (Baylis et al. 2008). Therefore, on what such fear and anxiety are based, social solidarity is needed to ensure there is a community’s distributive and fair principles. Thus, there will be no stigmatisation against covid-19 patients in self-isolation.

In Indonesia, publicity of social solidarity is marked by the emergence of the social solidarity movements in almost all regions in Indonesia. Based on the Focus Group Discussion (FGD) formulation by researchers, 28 participants stated that solidarity is the people’s biggest capital to survive and fight the pandemic. The public’s support determination for covid-19 patients who are undergoing self-isolation or survivors to survive shows that there is perception and threat felt by the public related to the crisis, satisfaction or dissatisfaction as an institutional response, long-standing trust, and response that is based on traditional values inherent as ideological orientation.

The existing forms of solidarity in the global community have spread, but only survive for a short time. Spontaneity in the mutual aid effort and voluntarily offering assistance when needed can be a constraint for continuation over a long period (Banulescu-Bogdan & Ahad, 2021). Facts show that a community with high social capital and trust can solve crises better than a community with no strong support network. This means that the communities are highly dependent on external parties.

## Mutual aid actions among the indonesian community

Amid COVID-19 emergency, most Indonesians are moved to help others infected by COVID-19 (Harbowo, 2020). According to Koentjaraningrat, mutual help is not only a spontaneous action to alleviate others’ crises, but the people feel they need each other, as reflected in communal work (Koentjaraningrat, 1985). Moreover, there is a shared understanding that COVID-19 is a “common enemy” that may threaten every individual’s safety, security, and tranquillity, strengthening the community’s solidarity actions (Wahyuningrum, 2020). This situation eventually brought the side effect of enhancing hygienic and healthy life values, respecting the environment, communal work, and empathy in the community. The values have culturally been an integral part of the Indonesians’ collective attitude. When the pandemic reached, the values became an essential strength in facing the pandemic together. Throughout the pandemic, the social bond is based on the sense of mutual aid for those in self-isolation with COVID-19, and is still potential, even showing an increasing tendency. Social solidarity, either organic or mechanical, is manifested in various actions of charity and volunteerism (Haryadi & Malitasari, 2020), either independently or collectively through community intermediaries, the COVID-19 Task Force, or the environment leader (head of neighbourhood unit [Rukun Tetangga/ RT] and Community Unit [Rukun Warga /RW]). Therefore, solidarity can be understood as the collective good of being bound together to help each other through what we perceive as a form of common interest (Tomasini, 2020: 235).

**Fig. 1:**
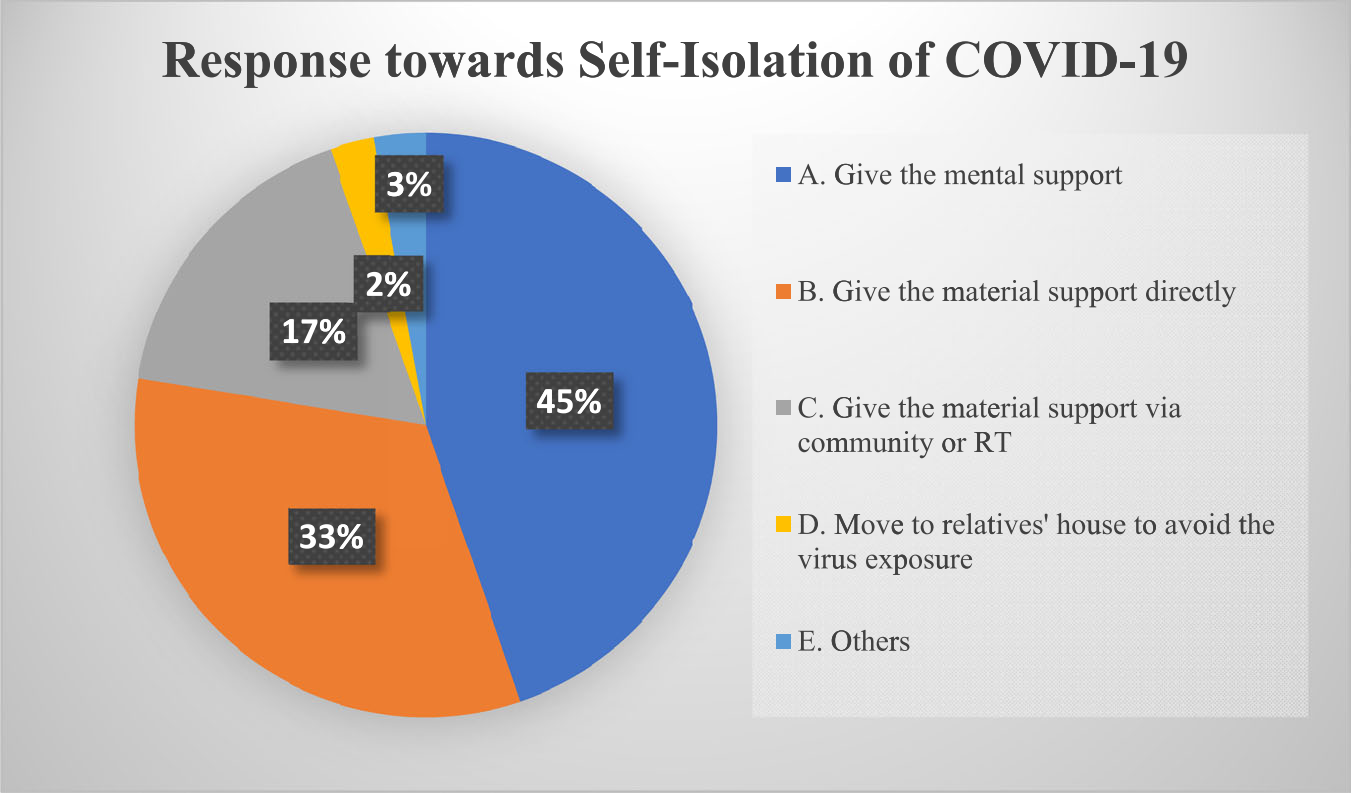
Response towards self-isolation of COVID-19. Illustrates the results of a survey conducted via Google Forms regarding responses when finding neighbours, friends, and relatives undergoing independent isolation at home. The survey was compiled from 445 participants representing 27 provinces in Indonesia. The survey results show that as many as 45% of participants provide moral support to remain enthusiastic during self-isolation through communication, suggestions and advice. 33% provided material assistance (groceries, food, vitamins, supplements, fruit) independently directly to those undergoing self-isolation. 17% provided material assistance (groceries, food, vitamins, supplements, fruit), organised collectively through the RT or community. 3% perform other actions, such as praying. The remaining 2% chose to move temporarily to a relative’s house or another place to avoid exposure.

Figure 1 is the survey result of 445 participants throughout Indonesia, there are various responses when they knew their neighbour, friend, and or relative were in self-isolation in their own house. 345 participants (45.5%) gave moral support and advice for the patients to keep fighting during self-isolation. One of the moral supports given by becoming someone to share and listening to their complaint through cellular call, so that they would not be sad or stressed with their health condition. This is as done by R, a volunteer of COVID-19 response in Kembanglimus Village, Borobudur District, Magelang, he is on standby until late night to listen to his neighbour’s and relatives’ complaints in their self-isolation (Rukmorini et al. 2021). The moral support for strengthening mental resilience and immunity during self-isolation is also given by the *Peduli Isolasi Mandiri Tegal* community through the I’m Care Up service. Accompaniment is given by survivors, motivators, child activists, who voluntarily registered to be volunteers.^1^ The moral support is given through WhatsApp, with both call and chat. The purpose of accompaniment is to mitigate psychological stress triggered by exposure to excessive information on the death rate risk of COVID-19 and speculation of possible conspiracy, which might worsen the pandemic. It caused anxiety, worry, and feeling of having their health threatened (Interview with HL, 21 August 2021). Maintaining mental health for those in self-isolation is also a special concern for WHO. Throughout the pandemic, WHO also gave psychological support for those in self-isolation worldwide through the mental health and psychosocial platform (UNICEF & WHO, 2021).

Public Health in the UK for instance, also advises people to maintain mental health by staying connected with others, helping and supporting others, talking about concerns, maintaining physical health, dealing with anger, maintaining daily routines and sleep patterns, and others (Herat, 2020). Concern for public mental health is also reflected in the Duke and Duchess of Cambridge’s support for the campaign. The Duke and Duchess of Cambridge’s Royal Foundation have even committed to allocating £1.8 million to support the frontline community and the nation’s mental health during COVID-19 (The Royal Foundation of The Duke and Duchess of Cambridge announces support for frontline workers and the nation’s mental health (2020)).

Besides moral assistance, 253 participants (33%) gave more concrete support of material form, including basic needs, food, vitamins, supplement, and fruits. Just like the action by AT in Lampung, he had the initiative to give material assistance to those in self-isolation. His assistance included rice, egg, noodle, vegetable, and fruit. He chose to assist in the form of basic needs, since they are the primary needs amid restricted space movement of those in self-isolation. In such a restricted condition, the food supply is crucial. He also intentionally gave them directly so those in self-isolation will receive them quickly, without any constraint or delay. This is the case considering the high death rate for COVID in Lampung. Lampung Province once had the highest case fatality rate (CFR) in Indonesia throughout 2021. Based on the data of the Ministry of Health from 1 January 2021 through 8 August 2021, the death rate in Lampung is 7.1 per cent (CNN Indonesia, 2021). The high death rate in Lampung encouraged its people to help each other. Even if the mutual aid action still tended to be partial and non-organised individual initiative, this presented a good indication that the social solidarity in Lampung had quite potential (Interview with AT, 12 September 2021).

MIM also shows a similar care in Makassar and YPT in Tebing Tinggi, that the two gave assistance in the form of vitamins and supplements to improve the stamina of those in self-isolation (Interview with MIM, Makassar, 12 September 2021; Interview with YPT, Tebing Tinggi, 12 September 2021). They understood that vitamins and supplements are useful to improve the immune system. The importance of vitamin in maintaining body immunity is stated in the research conducted by Kumar et al. (2021), even lacking vitamin and mineral in plasma concentration might lead to decreased performance of the body immune system, which is one of the constituents causing declined immune (Kumar et al. 2021). YPT, who often helps neighbours in self-isolation, argued that neighbours are the closest family that must be helped when facing a difficult situation, especially when in self-isolation and going out to have their needs is impossible. Therefore, it is a shared duty to help each other supply their daily needs in order to accelerate the recovery process (Interview with YPT, 12 September 2021). MIM also intentionally distributed the assistance directly so it would be more personal and right on the target. In Makassar, assistance distributed by the community is only concentrated in certain areas that are undoubtedly uneven (Interview with MIM, Makassar, 12 September 2021). Perhaps, the uneven distribution of assistance is also affected by geographic factors, such as area isolation, distance, and road condition (Damsar, 2016). The solidarity actions, both by MIM and YPT, show that it is empathy on which their altruistic actions are based. The purpose of such action is to alleviate others’ suffering, without expecting any reward. Therefore, individuals with altruism always attempt to consider others’ rights and welfare (Batson et al. 1981; de Waal, 2008).

Giving material assistance is also the authentic expression of care based on humanity, Even experiments have proven that altruism can give happiness and significance to those who accomplish it (Santi, 2021). Additionally, altruistic behaviour is an important contributor to well-being, health, and longevity (Soosai-Nathan et al. 2013). Just like what is done by TS in Tanjung Pinang. He felt deep happiness when he was able to help others in self-isolation, including neighbours, close friends, and even people who were not acquainted or of non-family relationships. It seems that the drive to do a good deed for others cannot be separated from the tendency of a deep feeling of the relationship with “all humans” as my in-group (McFarland et al. 2012). The assistance commonly given, including vitamins, food, and even child toys are directly put in front of the house of those in self-isolation while calling them via telephone to provide them with support from a distance. He often gave toys to children who are exposed to COVID-19 in order to maintain their mental health and keep them happy (Interview with TS, Tanjung Pinang, 12 September 2021). According to the United Nations Children’s Fund (UNICEF), fear and stress caused by COVID-19 can also increase children’s insecurity and may potentially cause more serious mental health problems if not dealt with promptly (UNICEF & WHO, 2021).

Material assistance distribution is also carried out with mediation by the environment or community leaders. Of 445 participants, 132 participants (17%) gave material assistance through an intermediary. According to one of the participants in Central Java, there are at least some special considerations of why the community became a relatively strategic medium to distribute assistance. First, assistance distribution could be given evenly. Thus, no person in self-isolation who did not receive any assistance or received too much assistance. Second, assistance is given in a scheduled manner and periodically. Third, the remaining assistance could be used as a reserve for others who might later be infected by COVID-19 and have to do self-isolation (Interview with RG, 12 September 2021).

Central Java is the prototype of a region with a high social solidarity movement. There is a local wisdom programme initiated by the Governor of Central Java, namely *Jogo Tonggo*, with the working principle based on humanity, communal work, involvement of all parties, non-permanent during an emergency, and transparency (Pocket book, “Community Based COVID-19 Response Acceleration Task Force at RW Level”, 2021). The essence of *Jogo Tonggo* is to work together to fight against distribution and transmission of COVID-19 and optimise social, health, and economic security network systems from the lowest level, namely RT, RW, and village/sub-district (Shodiq, 2021). Reflecting on the Central Java people’s characteristics, this approach is deemed more appropriate and more effective rather than large-scale social restrictions or regional lockdowns. However, this programme implementation is full of challenges, such as the people’s increasing mobility, and social-cultural dynamics making all activities less strict. Several studies have determined that there is a relationship between levels of understanding about COVID-19 prevention practices related to masquerading (Zhong et al. 2020). If the community understands the spread of COVID-19, applies prevention practices, and uses appropriate personal protective equipment, the risk of contracting COVID-19 can be prevented.

The people’s misperception of COVID-19 also worsened the situation. Getting infected by COVID-19 is often deemed a disgrace. Thus, people are afraid of having a test (Perdana, 2020). In its implementation, *Jogo Tonggo* operated well. RT leaders moved their people and raised funds to fulfil the family members’ daily needs exposed to COVID-19. This is under consideration that the whole family members must be under self-isolation. Female members in RT helped raising funds and handed them over to catering to order ready-to-eat food. The food is then distributed to the people in self-isolation. In 2020, there is assistance from *Jogo Tonggo* for those in self-isolation from the sub-district, which is raw food valued from Rp200,000. In addition, there is also assistance issued from RT treasury to supply ready-to-eat food to serve families in self-isolation (Forum Group Discussion with DRP, Head of RT, 12 August 2021). Actually, food cooking action for those in self-isolation has been a global movement, such as one in India, where through a home cook community named Aggarwal, the cooks cooked a large amount of food to be distributed to COVID-19 patients who are under self-isolation at home (Aljazeera.com, 2021).

Besides, through *Jogo Tonggo* as the medium, there is a community in Central Java that is driven to do collective action based on normative obligation for others, namely the *Semarang Semedulur* Community, a community which consider fraternity among the people who lives in Semarang. The initiators, with members of young doctors, intentionally took the initiative to help those in self-isolation. They had also been COVID-19 survivors. Thus, they understood well the condition of patients who are in self-isolation. To socialise this movement, the community members distributed pamphlets on social media networks (Instagram, Facebook, etc.) and welcomed the request for assistance in the form of vitamins, food, and basic needs.^2^

The spirit of mutual help, altruism, and care for others is inherent among the people in Ngablak Village, Ngablak District, Magelang Regency, Central Java. The majority of people in Ngablak village are vegetable farmers, and their care is manifested in performing farming activities on the abandoned lands since their owners are in self-isolation. The farmers, neighbours of the patients, usually moved fast, from spraying insecticides to harvesting vegetables and selling them in the nearby market. The result of selling the vegetables is then put on the doors of those in self-isolation. In addition, people also cut grass to feed the livestock of those in self-isolation. Since the COVID-19 pandemic, Ngablak Village established a group of volunteers with members distributed in seven sub-villages. The volunteers’ duties are, among others, distributing food and vitamins to patients in self-isolation. The assistance or solidarity did not only come from COVID-19 Task Force members, but also from nearby neighbours. The assistance of harvesting vegetables and cutting grass must be done so that the farmers exposed to COVID-19 would undergo self-isolation with a peaceful mind (Rukmorini et al. 2021). Central Java is indeed a region with a high level of social solidarity. Based on the questionnaire, 54 out of 65 participants decided to help those in self-isolation since such an action is part of their social obligations.

The solidarity atmosphere is also felt in Cipageran, West Java, where people help those in self-isolation. They give assistance voluntarily, from donating money to foodstuff, as one conducted for M (45), a person infected by COVID-19. The people cooked food and delivered it every morning to the M’s house with the other four family members. The assistance is distributed to M in compliance with the health protocol, including wearing a mask and gloves. The humanity solidarity in Cipageran even broke the boundaries of difference, since M is a Christian who lived in an environment of mostly Muslims. It is clear that helping others is carried out regardless of the difference, either based on tribe, race, or religion (Sinaga, 2020).

### Giving assistance as communal solidarity

At the time of Covid, social solidarity is shown by community initiatives at the community level to perform self-isolation, both related to health, security, and communal comfort which is entitled to “community lockdown”. The community consciously participates in assisting in various forms, including disinfectants, distributing masks, and hand sanitisers, and actively supporting campaign Healthy Community Movement (GERMAS). Yet, it is not to mention socio-economic-based humanitarian movements, ranging from charity to social security, in the form of medical and vitamin assistance, food supply, subsidies for vulnerable groups, solidarity with salary cuts and others, and solidarity for helping patients.

In Banjarmasin, South Kalimantan, the city government also gave social assistance to 1000 people in self-isolation. This movement is conducted by the local government, starting from recording the data per family head and each individual. In addition, there are *Relawan Makkah*, *Sahabat Yatim*, which distributed food assistance through couriers, such as Grab and Gojek. The volunteers are from Banjarmasin and Banjarbaru, a region with PPKM Level 4 status. However, the Banjarmasin people, especially in inland areas, tended to have a closed character, thus they did not inform their health condition to village officials and did not check up with the doctor. Such a situation is worsened by the health facilities that are limited and located distant from their settlement. Finally, the only solution is to isolate one village to break the virus transmission chain, like one in upstream of the river, Papagaran. The limited medical instruments also drove surrounding people to seek alternative treatment by consuming herbal medicine (*buin pasu*) which was obtained from the forest (Forum Group Discussion with M, 9 August 2021).

In the context of administering medicines and vitamins, social preference for the types of medicines needed vary. According to the analysis, the choice to use herbal has no relationship with the level of education, this is as evidenced in the results of the Chi-Square statistical test that the ordinal data correlation coefficient is 0.059 and the sig value is 0.213 which is greater than 0.05. It shows that there is no relationship between education and the rationality of drug consumption. Determination of the rationality of drug selection is based on three categories, namely very rational people who choose a combination of chemical and herbal medicines; the rational category is determined from the use of chemical drugs only; while less rational only rely on herbal medicines (as can be seen in Table 1).

**Table 1:** Relations of educational level with drug consumption during Covid-19 using cross-tabulations.

			Drug consumption			
			Less rational	rational	Very rational	Total
Latest education level	Elementary school	Count	0	0	1	1
		% within educational level	0.0%	0.0%	100.0%	100.0%
		% within drugs consumption	0.0%	0.0%	0.3%	0.2%
		% of Total	0.0%	0.0%	0.2%	0.2%
	Junior high school	Count	0	0	1	1
		% within educational level	0.0%	0.0%	100.0%	100.0%
		% within drugs consumption	0.0%	0.0%	0.3%	0.2%
		% of Total	0.0%	0.0%	0.2%	0.2%
	Senior high school	Count	13	9	91	113
		% within educational level	11.5%	8.0%	80.5%	100.0%
		% within drugs consumption	26.5%	28.1%	25.0%	25.4%
		% of Total	2.9%	2.0%	20.4%	25.4%
	Bachelor	Count	15	15	100	130
		% within educational level	11.5%	11.5%	76.9%	100.0%
		% within drugs consumption	30.6%	46.9%	27.5%	29.2%
		% of Total	3.4%	3.4%	22.5%	29.2%
	Master	Count	18	5	135	158
		% within educational level	11.4%	3.2%	85.4%	100.0%
		% within drugs consumption	36.7%	15.6%	37.1%	35.5%
		% of Total	4.0%	1.1%	30.3%	35.5%
	Doctoral	Count	3	3	36	42
		% within educational level	7.1%	7.1%	85.7%	100.0%
		% within drugs consumption	6.1%	9.4%	9.9%	9.4%
		% of Total	0.7%	0.7%	8.1%	9.4%
Total		Count	49	32	364	445
		% within educational level	11.0%	7.2%	81.8%	100.0%
		% within drugs consumption	100.0%	100.0%	100.0%	100.0%
		% of Total	11.0%	7.2%	81.8%	100.0%

According to Table 1, the highest ratio of drug consumption under very rational category came from participants with elementary and junior high school education, each 100%. The highest ratio of drug consumption under the rational category came from participants with an undergraduate degree (11.5%). The highest ratio of drug consumption in the less rational category came from participants with high school education, each 11.5%.

In East Nusa Tenggara, South Timor Tengah Regency, a church community paid more attention to the congregation infected by COVID-19, and the church regularly distributed assistance in the form of basic needs. Similar action also took place in Medan, where a church community actively gave a *doakonia* service, service of love (table), to those in self-isolation. Material support in the form of basic needs is usually obtained from a donation in the form of money from donors from Rp50,000–Rp3,000,000 and distributed in the form of basic needs to the congregation (Interview with RLT, 12 September 2021).

Related to the residential places of the participants, namely those who come from urban, rural/agrarian, and coastal areas. Interestingly, the survey data processed using Cross-Tabulations and chi-square analysis, it shows that the highest solidarity level to help participants comes from participants who live in urban areas (11.6%); participants coming from rural or agrarian participants tend to be moderate (20.0%). Meanwhile, the lowest solidarity level came from participants who lived in the coastal areas (37.5%) (it can be seen in Table 2).

**Table 2:** Relations of geographical locations and level of solidarity using cross-tabulation and chi-square analysis.

			Helping community					
			Very low	Low	Moderate	High	Very high	Total
Community	Urban	Count	83	22	111	88	40	344
		% within community	24.1%	6.4%	32.3%	25.6%	11.6%	100.0%
		% within helping community	74.8%	55.0%	85.4%	80.7%	72.7%	77.3%
		% of Total	18.7%	4.9%	24.9%	19.8%	9.0%	77.3%
	Rural or agrarian	Count	22	16	17	17	13	85
		% within community	25.9%	18.8%	20.0%	20.0%	15.3%	100.0%
		% within helping community	19.8%	40.0%	13.1%	15.6%	23.6%	19.1%
		% of Total	4.9%	3.6%	3.8%	3.8%	2.9%	19.1%
	Coastal	Count	6	2	2	4	2	16
		% within community	37.5%	12.5%	12.5%	25.0%	12.5%	100.0%
		% within helping community	5.4%	5.0%	1.5%	3.7%	3.6%	3.6%
		% of Total	1.3%	0.4%	0.4%	0.9%	0.4%	3.6%
Total		Count	111	40	130	109	55	445
		% within community	24.9%	9.0%	29.2%	24.5%	12.4%	100.0%
		% within helping community	100.0%	100.0%	100.0%	100.0%	100.0%	100.0%
		% of Total	24.9%	9.0%	29.2%	24.5%	12.4%	100.0%

	Chi-square tests		
	Value	df	Asymp. Sig. (two-sided)
Pearson Chi-square	19.781	8	.011
Likelihood ratio	18.401	8	.018
Linear-by-linear association	1.454	1	.228
No. of valid cases	445		

Cells (33.3%) are expected to count less than 5. The minimum expected count is 1.4.

The sig value of 0.01 is smaller than 0.05, which implies a significant.

## Communal work as the basis of social solidarity in the time of pandemic

Communal work is a social capital that can still be relied on to solve difficulties in daily life in Indonesia. The Legatum Prosperity Index 2020 stated that Indonesia ranked sixth for social capital, covering the strength of personal and social relationships, trust in the institution, social norms, and civil participation (Harbowo, 2020). Communal work has placed Indonesia in the highest order on the World Giving Index of the Charities Aid Foundation (CAF). Before the pandemic, in the World Giving Index 2018, Indonesia ranked first (with a score 59%) in terms of helping foreigners 46%, frequency of money donations 78% and becoming a volunteer 53% (*Caf World Giving Index*, 2018). During COVID-19 pandemic, based on the World Giving Index 2021, Indonesia also ranked first with a score of 69%, covering helping foreigners 65%, frequency of money donations 83%, and becoming volunteers 60%. Such achievement made Indonesia to be the most generous country in the world and a country with thrice more volunteer rate than the global average (World Giving Index 2021).

Following Lin, social capital refers to resources instilled in a social structure that is accessed and/or mobilised in purposeful action. Thus, social capital is rooted in social networks and social relations. The resource is not limited to material goods needed to support and improve human life, but also covers symbolic resources. Social capital should benefit an individual who acts for a purpose. In this context, interaction is deemed the medium to achieve the purpose of action (Lin et al. 2001, 29-41).

Communal work in Indonesian society generally covers two categories, namely mutual aid and civic service. Mutual aid refers to reciprocal communal activities, such as giving assistance to neighbours who are having a disaster such as death of a family member, building a house or big work related to human life cycle. Mutual help means giving and philanthropic, ad hoc, and the recipient is an individual. Meanwhile, civic service refers to working together for people’s interest, such as repairing roads, irrigation channels or other public facilities. What is unique in civic service is that the target recipient is the public and the activity is carried out in a structured manner. Besides mutual aid and civic service, there is another category, that is the spirit of communal work, that is not manifested in the form of activity, but in a valuing system on which the activity is based. Koentjaraningrat calls it *gotong royong* [mutual cooperation]. Most communities in the world have the spirit of communal work. Thus, it is not addressed to a particular community, but even also to a community that is often associated with individualism (Koentjaraningrat, 1971, 1985).

The founding fathers of Indonesia made communal work the *Weltanschauung* for independent Indonesia. In the session of *Dokuritsu Zyunbi Tyoosakai* (Investigating Committee for Preparatory Work for Independence, BPUPKI) on 1 June 1945, Soekarno expressed his ideas of the five bases of Indonesia called Pancasila. The five bases could be “squeezed” into *Trisila* [3 bases], namely socio-nationalism (sense of nationality and humanity), socio-democracy [sense of social justice and democracy], and divinity. *Trisila* can then be “re-squeezed” into *Eksasila* [one basis], namely communal work. In Soekarno’s opinion, communal work means working together which reflects the awareness that all citizens have equal rights and obligations. He confirmed that communal work is an Indonesian original term that describes the Indonesians’ pure souls. With this concept, Soekarno wanted to form Indonesia as an “all for all” country. Soekarno’s ideas are principally acceptable to and supported by other nationalists like Soepomo and Muhammad Hatta. Soepomo promoted the idea of an integral country where the country was united with its entire people and solved any classes in the field, which was covered by the spirit of communal work and the family spirit. Meanwhile, Hatta stated that the establishment of the new country was based on collectivism defined as communal work and joint effort (Dewantara, 2017).

Communal work in the founding fathers’ rhetoric referred more to the general ethos or the spirit of mutual aid, and reflected the original idea of moral obligation and general reciprocity. Communal work, in this definition has been accepted as part of the national culture and, therefore, is also deemed as part of the definition of the Indonesians. In its development, the term *gotong royong* [communal work] is taken and reworked by the state as a cultural-ideological instrument to mobilise workers. This term is later used to refer to certain forms of worker service. Foreigners will describe these forms of worker service as communal work, while the directly involved actors label as worker demand made by the state (Bowen, 1986). Communal work for various forms of worker service had been used since the Dutch East Indies colonial era and the Japanese occupation. After Indonesia’s independence, the government made communal work as a form of social engineering and smart linguistic strategy where the elites regulated the control over citizenship of formation. The term communal work is often used as a political instrument to raise citizens’ sense of belonging and collective compliance to mobilise their participation in and contribution to development programmes implementation. Communal work that had been co-opted into the nation-state development discourse changed the popular view of worker and capital mobilisation that were previously moral obligations and general reciprocity to be submitted (Suwignyo, 2019).

State-driven communal work is not necessarily bad. In certain situations, this is even useful to invigorate social solidarity and the spirit of mutual aid, especially during difficult times like economic crises, the recovery after natural disasters, and outbreaks. The people define the state’s existence, both the central government and local government of various levels, as the form of great care and appreciation of cultural uniqueness and local wisdom. Those who feel the government pay them attention are encouraged to participate in communal work activity (Titus, 2008). With regard to communal work in COVID-19 pandemic response, an interesting case example is from Central Java through *Jogo Tonggo*” including helping those who are in self-isolation.

The Provincial Government of Central Java had given special attention to this issue by forming the *Jogo Tonggo* Task Force. The people directly facing COVID-19 transmission are taken as the vanguard in dealing with COVID-19 in a systematic, structured, and whole manner. This policy is a community-based COVID-19 pandemic response acceleration effort up to RW level (Instruction of Governor of Central Java No. 1 of 2020 on Community Empowerment in COVID-19 Response Acceleration at Neighbourhood Council Level through Formation of *Jogo Tonggo* Task Force”). The Provincial Government Central Java had also published a pocket book intended to be technical guidelines on implementing *Jogo Tonggo*. The book explained that the *Jogo Tonggo* policy is an effort to encourage various elements of the community’s active participation in keeping themselves from COVID-19 transmission, mutual concern and care, and not giving any bad stigma to those positively exposed to Coronavirus. The effort to encourage the various elements of community’s active participation is carried out by involving and synergising the role of various parties, including community members, village midwife, Community Protection Unit [Perlindungan Masyarakat], Expected Family Programme [Program Keluarga Harapan], Field Farming Extension Worker [Penyuluh Pertanian Lapangan], village facilitators, youth organization, *dasa wisma*, neighbourhood health centre, and other local organisations (Central Java Official Portal, 2021a). The *Jogo Tonggo* programme has been acknowledged as one form of public service innovation during the pandemic by the Central Government through the Ministry of Empowerment of State Apparatus and Bureaucracy Reform. This can be examined from the award received by *Jogo Tonggo* as the Best Innovation of COVID-19 and Complaint Response 2020 for the COVID-19 Response Public Service category (Central Java Official Portal, 2021a; CentralJava Official Portal, 2021b).

Local governments’ policies related to the COVID-19 pandemic have been critically responded to by some researchers, since the COVID-19 pandemic response in Indonesia was initially centralised. However, the central government did not point out further measures to actively lead and coordinate the national response to deal with this pandemic (Morris, 2021). The central government focused more on the mitigation of the pandemic’s economic impacts. Besides being slow and inadequate, the national health response is also often incompetent. It can be observed from the increasing crisis during the pandemic in early 2021, where the hospitals throughout Java and Bali are full and the number of positive the COVID-19 cases and deaths daily increased rapidly. The central government’s unsuccessful effort in its centralised handling made local governments and the people assume most of the pandemic burden (Meckelburg, 2021). Does this mean communal work moved by local governments is the distribution of consequence for the central government’s incapability in the centralised handling of COVID-19 outbreak and further consequence of the use of communal work as a cultural-ideological instrument? The fact is unnecessarily so. There is still communal work that reflects moral obligation and general reciprocity. This could be observed, for example, from the willingness of about 15000 medicine students from 158 colleges who registered as a volunteer in the effort to fight COVID-19 (Galiya, 2020). Through FGD, it is found that some informants decided to be volunteers since they are moved to help others who confirmed positive for COVID-19 and must carry out self-isolation. Meanwhile, survivor status stated that they received assistance like fruit, foodstuff and drink, and vitamins from colleagues, neighbours, and families during self-isolation. The survey results also show that the government, through the bureaucratic network up to the lowest level (RT/RW) plays essential roles in coordinating and distributing assistance to patients in self-isolation both at home and in the place provided by the government. The growth of awareness and spirit to do communal work to help COVID-19 patients is undoubtedly also influenced by religion. As stated by Seda et al. (2020), trust in religious figures, and benefits obtained from religious identity and from religious institutions and groups are still relied on to deal with disaster and outbreak response in daily life (Seda et al. 2020).

## The role of neighbourhood association to limit virus transmission

Restricting people’s mobility is one of the non-pharmacological efforts aimed at reducing social interaction between humans and ultimately stopping virus transmission. Human mobility is a paramount factor influencing virus transmission (Changruenngam et al. 2020). In the United States, movement patterns are closely correlated with increases in the subpopulation of COVID-19 cases (Badr et al. 2020). Not all groups are at equal risk of exposure to the COVID-19 virus, and in Ireland and elsewhere, the impact of COVID-19 is associated with a minority of group membership. Disadvantaged ethnic minorities and immigrants are infected with this virus to a greater extent than the total population; partly due to financial condition and place of residence. For example, people who live in confined spaces, work in retail with low wages, have no private transportation and have to work outside the home, have a higher risk of exposure to the virus (Foran et al. 2021).

Thus, limiting people’s mobility is an effective and efficient way to contain virus transmission. The transmission of COVID-19 can be narrowed down through various procedures, such as limiting human activities that invite crowds, closing public places, imposing lockdowns, working and studying from home, physical distancing, and self-isolation. In the Indonesian context, cohesiveness, social solidarity, and cultural values manifest in *gotong royong* among people, especially when self-isolation is one factor that accelerates healing. Various assistance distributed by the social environment to residents undergoing independent isolation also prevents patients from going out for activities to look for food or vitamins. In addition, the health workers, in this case, the village midwife and the COVID-19 Task Force, are quite proactive in providing health assistance so that the condition of self-isolation patients is adequately monitored.

The spread of Covid-19 could be influenced by sociocultural factors such as poor sanitation in certain areas, places of worship which remain open, crowds at entertainment venues at night, etc (Purwanto et al. 2021). Policies that take into account an increase in case intensity, for example the physical distancing policy through the Community Activity Restrictions (PPKM) programme, which has experienced an increase in cases, implements a more stringent physical distancing policy than areas that have experienced an increase in cases. The success of PPKM implementation has also been determined at the semi-formal bureaucratic level.

The ‘semi-formal’ bureaucracy includes an RT institution and a higher institution of RW. They are called ‘semi-formal’ since RT and RW institutions are not formal bureaucracies to which officials are assigned as state civil apparatus and obtain salaries. Despite their informal status, RT and RW are under the bureaucratic authority of *lurah* or village head. These institutions can even be used as the vanguard of bureaucracy that directly faces the community members. Similarly, during the Covid-19 pademic, RT and RW can be used to solve such problems. RT/RW has become at the forefront of government efforts to manage the COVID-19 crisis. President Joko Widodo has identified neighbourhood leaders as “the key” to control the pandemic, tasking them with enforcing mobility restrictions in their areas and facilitating the distribution of economic assistance to eligible residents.

Historically, the RT and RW heads are chosen by the people in their environment. Thus, the position of RT and RW is more legitimate in front of the people. Therefore, RT and RW institutions played significant roles during the Independence War and in later periods, not only in administrative matters, but more prominent roles in terms of community security, especially in times of emergency. Such significant roles are institutionalised and strengthened by the Indonesian government because of their practical and strategic functions in all social and political fields. Various kinds of government programmes (aids) can be a success when socialised well until RT cells. Similarly, the role of RT has been very significant in response to and dealing with COVID-19 Pandemic in the last two years since 2020.

The role of RT and RW started to show in the early stage of dealing with COVID-19 cases, which became the early detection of people who were infected with the virus. This is mainly to know that any residents under RT who conduct self-isolation. Out of the 345 participants, 24.7% of participants get informed about the people who conduct self-isolation obtained from RT/RW. Meanwhile, the information comes from the neighbour (19.1%). An interesting point that exposes the RT’s significant role is that when resident got infected with COVID-19, they did not report to the hospital, but they (55.9%) first reported to the COVID-19 Task Force whom also consists of the RT Head and the parties from the Community Health Centre. Meanwhile, only 7.2% inform the neighbours. It means that they still have high trust in RT institutions.

Furthermore, RT Institution also had a significant role in giving help to people infected by COVID-19. Those in self-isolation are not allowed to go out and connect with health community members. Therefore, all of their needs must be given by others so that they would remain at home or a place of self-isolation. In this case, the RT institution is still trusted by the people who are part of the RT environment to coordinate other healthy and capable people’s assistance. 24% of informants trusted RT institutions to assist and handle the needs of COVID-19 survivors in self-isolation. There are various types of assistance given to COVID-19 patients in self-isolation, including: food, drink, fruit, vegetable, vitamin, medicine, and various necessities needed by COVID-19 patients.

## Conclusions

In order to deal with this pandemic that has a global impact on all lines of life, the implementation of social solidarity in traditional societal values, especially mutual cooperation [*gotong royong*] is the basis of a strategy to survive during this pandemic. The mutual cooperation eventually becomes a way of life of society as it has been manifested by solidarity actions which have been performed on both direct and virtual platforms. Those solidarity actions have been passed down from generations of the Indonesian people. It has easy inspired people to create similar actions. Therefore, total solidarity could be developed naturally in our society. In addition, the semi-formal institution, in this regard RT/RW, fosters COVID-19 inhabitants who conduct self-isolation to recover from the depression due to the pandemic. It is in response to handling the public needs, which the government has yet not met, so returning to the traditional values of society in the form of mutual cooperation, is an alternative way to overcome social chaos. The solidarity actions are performed by people in all corners of the country, both in rural and urban areas, ignoring social, religious, cultural, and educational backgrounds. According to our survey, the major reason for performing these actions is to encourage humanism. The solidarity programmes run and flow naturally without obstacles from society. The active participation of the community in handling the impact of the COVID-19 pandemic has supported the effectiveness of solidarity programmes. The value of solidarity embodied in *gotong royong* has become a habitual tradition of some members of society. This pandemic has tested the solidness and strength of the community, it shows that they are able to survive in the midst of any other difficult situations.

## Data Availability

The datasets generated during and/or analysed during the current study are not publicly available due to restrictions containing information that could compromise the privacy of research participants. However, the datasets are available from the corresponding author upon reasonable request.

## Footnotes

1 There are at least 6 volunteers joining, both from Tegal Regency and outside.

2 Potential recipients of assistance could request for assistance and preference of type of assistance through the mobile number written. This assistance is prioritised for those in self-isolation with criteria affected by PPKM, such as losing their job or closing of business. In addition, they are also required to attach a photo of identity card and antigen/PCR test letter with positive results. The assistance is directly distributed by the community members. When in a family there is only one person in self-isolation, the assistance is given daily at a value ranging from Rp30,000–Rp50,000. It is different if the whole family members are in self-isolation, that the assistance is given regularly, once a week, at a value ranging from Rp300,000–Rp400,000. The assistance given for 14 days or more pursuant to the health development. Most of the source of funds from this movement is from donors. The types of assistance given varied, such as cash ranging from Rp200,000–Rp500,000 per donor, basic needs, rice, noodle, snack, medicine, and fruit (Interview with GH, 21 August 2021).
